# Juvenile fish assemblages in temperate rocky reefs are shaped by the presence of macro-algae canopy and its three-dimensional structure

**DOI:** 10.1038/s41598-017-15291-y

**Published:** 2017-11-07

**Authors:** Adrien Cheminée, Jérémy Pastor, Olivier Bianchimani, Pierre Thiriet, Enric Sala, Jean-Michel Cottalorda, Jean-Marie Dominici, Pierre Lejeune, Patrice Francour

**Affiliations:** 1 Université Côte d’Azur, CNRS, FRE 3729 ECOMERS, Nice, 06108 France; 2 Univ. Perpignan Via Domitia, Centre de Formation et de Recherche sur les Environnements Méditerranéens, UMR 5110, 66860 Perpignan, France; 3 CNRS, Centre de Formation et de Recherche sur les Environnements Méditerranéens, UMR 5110, 66860 Perpignan, France; 4 Septentrion Environnement, Marseille, 13008 France; 5 Muséum National d’Histoire Naturelle, UMR 7208 BOREA, Station Marine de Dinard - CRESCO, 35800 Dinard, France; 6 National Geographic Society, Washington DC, 20036-4688 USA; 7 Réserve Naturelle de Scandola, PNR de Corse, Ajaccio, 20184 Corse France; 8 STARESO, Station de Recherches Océanographiques et Sous-Marines, Calvi, 20260 Corse France

## Abstract

Arborescent macro-algae forests covering temperate rocky reefs are a known habitat for juvenile fishes. However, in the Mediterranean, these forests are undergoing severe transformations due to pressures from global change. In our study, juvenile fish assemblages differed between pristine arborescent forests (*Cystoseira brachycarpa* var. *balearica*) *versus* an alternate state: bushland (Dictyotales – Sphacelariales). Forests hosted richer and three-fold more abundant juvenile assemblages. This was consistent through space, whatever the local environmental conditions, along 40 km of NW Mediterranean subtidal rocky shores (Corsica, France). Among *Cystoseira* forests, juvenile assemblages varied through space (i.e. between localities, zones or sites) in terms of total abundance, composition, richness and taxa-specific patterns. More than half of this variability was explained by forest descriptors, namely small variations in canopy structure and/or depth. Our results provide essential cues for understanding and managing coastal habitats and fish populations. Further studies are needed to explain the residual part of the spatial variability of juvenile fish assemblages and to help focus conservation efforts.

## Introduction

In community ecology, whether terrestrial or marine, environmental drivers may act at multiple and nested spatio-temporal scales to shape communities. At global spatial scale, structural and functional connectivity is known to shape populations through, for example, migrations of adult individuals between remote localities or the dispersal of reproductive products^1,2^. At intermediate scale, the configuration of a stretch of coast and putative consequences for local ecosystem functioning may in addition shape assemblages (e.g. sheltered *versus* exposed coast^3^). At local scale, between sites in a given habitat or between different habitats at a given site, residual variability in communities may be explained by differences in three-dimensional structure which determine habitat ‘quality’ in terms of the ratio between food availability and the predation rate, resulting in active habitat choice and/or differential mortality of individuals between habitats^4^. Habitat structure may be defined as the amount, composition and three-dimensional arrangement of the physical components (both abiotic and biotic) at a location.

In the case of marine fishes, previous studies have highlighted the importance of these various spatial scales. In the Mediterranean, some studies highlighted the importance of the dispersal capacities of fishes at the egg, larval, juvenile and recruit stages^5–7^. Regarding habitat characteristics, complex habitats formed by macrophytes have been proved to host more abundant and/or more diversified communities compared to simpler habitats^8^. More specifically, habitats with complex and/or heterogeneous three-dimensional structure are believed to improve survival for juvenile fishes, notably by providing refuge against predators^9^. There is therefore rising concern worldwide regarding habitat transformations under anthropogenic pressures. It has been observed that seagrass meadows and ‘macro-algae’ (i.e. Multicellular Photosynthetic Organisms belonging to the Chlorobionta, Rhodobionta and Phaeophyceae) forests are being replaced by less complex habitats^10^. Such habitat shifts are due to various factors and their possible synergetic effects, such as water pollution^11^, invasive species^12^, overfishing and resulting trophic cascades^13^, or physical disturbances such as trampling^14,15^. These shifts may have a dramatic impact on associated communities. They may alter their’habitat quality’, as defined above, which may in turn compromise their functioning as, for example, spawning or nursery grounds. Habitat complexity (e.g. meadow density) or heterogeneity (e.g. patchiness) have been demonstrated to drive juvenile fish abundance patterns^16–18^. In the Mediterranean, recent studies have focused on the decline of formerly abundant macro-algae forests formed by the genus *Cystoseira* (Fucales). These canopy-forming species used to be dominant in Mediterranean rocky reefs^15^. Their complex three-dimensional structure is recognized to host high biodiversity^15,19^. When disturbances lead to community shifts, *Cystoseira* forests are replaced by less complex alternate stable states without canopy and dominated by less complex and non-perennial macro-algae (notably Dictyotales and Sphacelariales bushland), turf algae, or sea urchin barrens with encrusting Corallinaceae^10,15,20^. However, very few studies have investigated the consequences of these shifts for associated communities, notably for adult or juvenile fish assemblages. In a previous study by Cheminée *et al*.^21^, carried out one year before the present study at only one of the 20 study sites used here, authors showed that Fucales forests of *Cystoseira brachycarpa* J. Agardh var. *balearica* (Sauvageau) Giaccone (hereafter *C. balearica*) hosted more abundant populations of the wrasses *Symphodus roissali*, *S. ocellatus* and *S. tinca*, when compared to the less structurally complex bushland of Dictyotales and Sphacelariales (DS). However, this study was restricted to only one site in Corsica (NW Mediterranean) and few fish species. The consistency of these results had to be confirmed through time and space (another year, and at a greater number of sites), and considering the full necto-benthic juvenile assemblage of the habitat. This information is important as a basis for conclusions regarding the potential nursery role of such forested habitats in the context of their conservation.

**Figure 1:**
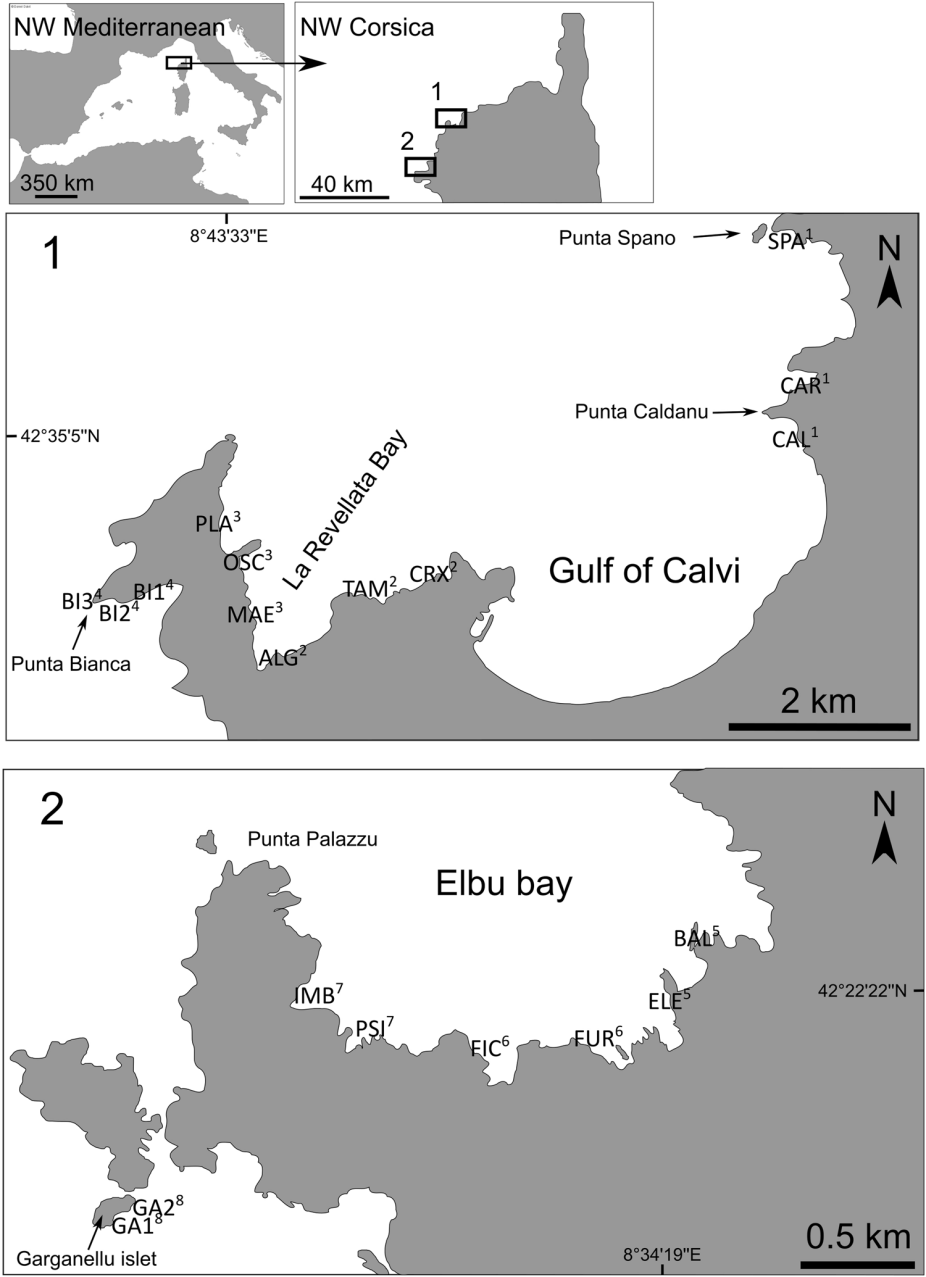
Studied zones and sites at each locality. For each locality (1: La Revellata; 2: Scandola) study sites are indicated in capital letters. Superscript Arabic numbers indicate repartition of sites into zones (1 to 4: within La Revellata locality; 5 to 8 within Scandola locality). The map was drawn using free and open source software Inkscape 0.91 (https://inkscape.org/en/) and QGIS 2.14 (http://www.qgis.org/). Map was drawn by authors using online Standard tile layer from OpenStreetMap data as background model (© OpenStreetMap contributors), available under ODbL licence (CC-BY-SA) at http://www.openstreetmap.org/.

**Figure 2:**
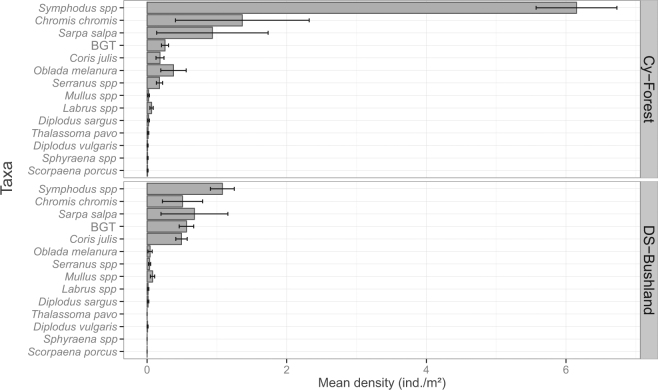
The mean juvenile assemblage in *Cystoseira balearica* forests (Cy-Forest) differed significantly from the one observed in Dictyotales-Sphacelariales (DS) bushland (PERMANOVA, see results). For each habitat, barplots display the mean density of each taxa and its standard error (SE). See taxa details in Table 1.

Accordingly, the aim of the present study was to test two hypotheses regarding spatial variations of juvenile fish assemblage structure, which was analyzed using both multivariate (taxonomic composition and density) and univariate descriptors (total juvenile fish density and taxonomic richness): (1) structure of juvenile fish assemblages differs between arborescent forests (*C. balearica*) and bushland (Dictyotales – Sphacelariales) consistently through space (among coastal sites scattered over 40 km, Fig. 1); (2) focusing on juvenile fish assemblages in arborescent forests, juvenile fish assemblage structure is affected by the depth and the three-dimensional structure of the forest (canopy cover and height).

## Material and methods

### Ethics statement

The observational protocol was submitted to the regional authority ‘Direction interrégionnale de la mer Méditerranée’ (the French administration in charge of Maritime Affairs), which did not require a special permit since no extractive sampling or animal manipulations were performed (only visual censuses in natural habitats), since the study did not involve endangered or protected species and since the surveyed locations were not privately owned.

### Study sites and sampling methods

In the Mediterranean, due to the decline of *Cystoseira* forests^15^, surveys must be conducted in a study area were these forests still occur, such as Corsica Island (NW Mediterranean). To test our two main hypotheses, we sampled juvenile fish assemblage structure (and some environmental variables as covariates) in forest and bushland, at 20 sites (i.e. both habitats were sampled at each site). To select sampling sites, we randomly defined 2 localities within the study area: La Revellata and Scandola (Fig. 1). Within each locality, we randomly defined 4 zones, and within each zone we randomly defined 2 to 4 Sites. The localities are 40 km apart from each other, along the western coast of Corsica. They both display rocky shores with large stands of subtidal (0–15 meters) forests of *Cystoseira balearica* ^22,23^. It is worth noting that the ELE site in Elbu Bay was previously studied in 2009^21^. First, in order to select comparable study sites, two observers (AC, JP) systematically explored by kayak, snorkeling and free-diving the shallow (1–10 meters) habitats along the 5 km coastline of Elbu Bay and the 5 km coastline of la Revellata Bay (Fig. 1). We schematically mapped the micro-habitats of the entire explored area (substrate type, biotic cover, slope, depth)^24^. This micro-habitat localization was completed through personal communications with Mediterranean phycologists or experts (K. Ballesteros, J.-M. Dominici, P. Lejeune, C. Pelaprat, and A. Chery). On the basis of this data we randomly selected for each locality a set of sites each containing both habitat types, i.e. wide *C. balearica* forests and DS bushland (as described in Cheminée *et al*.^21^). Within each site, for each habitat, 7 replicates of 1 m² (see below) were randomly selected so that percent cover of macrophytes was above 60% (for replicates in *C. balearica*) and 40% (for replicates in DS). Random replicates were realized between 1 and 8 meters depth, and all other micro-habitat biotic and abiotic characteristics were kept as constant as possible (i.e. gentle 0–23° slope, only continuous rocky substrate without crevices). From July 14^th^ to August 8^th^ 2010, during daylight (between 10 am and 4 pm), at each site, for all replicates in both habitat, the same SCUBA divers, previously inter-calibrated, performed 5 minute underwater visual censuses (UVC) of juvenile fishes, as described in other works^17,21,25^. Rough sea and poor visibility days were avoided. The divers recorded the abundance and size per taxa of benthic juveniles observed within each 1 m² plot. Sampling was performed during the known settlement or post-settlement period of many Mediterranean species^26–29^. This period also follows the annual biomass maximum for most subtidal macroalgal assemblages and more specifically *Cystoseira* species^23,30^. According to previous studies^21,27^, the juvenile fish families we expected in these habitats belonged mostly to the Labridae and Serranidae families. Nevertheless, our sampling took into account all necto-benthic and crypto-benthic juvenile species encountered. This sampling method was well-suited to our objectives since the studied species are sedentary when juvenile, and similar size and numbers of replicates were proven to provide accurate density data in other works^17,21,24,25^. The total length (TL) of individuals ( ± 0.5 cm) was estimated by means of fish silhouettes of different sizes on a submersible slate^27,31–34^. For most rocky reef fishes in the Mediterranean, size at settlement is around 10 mm TL^27,29^. Since our sampling was done at mid-point of the known settlement time frame of the studied taxa, and because the settlement schedule of different species may display differences^27–29^, our visual censuses took into account the young of the year (juveniles, y_0_ individuals) and also size-classes that might correspond to young of the previous year (y_+1_) (Table 1). The basis for categorizing fish as juveniles was as follows: for taxa with detailed previous studies on reproduction schedule, settlement timing, size at settlement and growth (e.g. Labridae, Serranidae and Sparidae), y_0_ + y_+1_ upper size limit was taken from literature^24,28,29,33,35^. Otherwise (e.g. Blenniidae), all individuals smaller than one third of adult maximum total length^26^ were considered as juveniles, as in previous works^17,36^. To determine habitat characteristics, depth (meters), canopy height (cm) and cover (%) were recorded in each replicate plot. For the forest, canopy height and cover were then used to calculate a single descriptor (“volume” (cm^3^)) to quantify the canopy’s three-dimensional structure.

**Table 1:** Observed juvenile assemblage in both habitats.

Family	Species	Taxa abr.	y_0_ + y_+1_ upper limit	*C. balearica* forest		DS bushland		Contrib. (%)	Cum. (%)
				Mean	se	Mean	se		
Labridae	*Symphodus* spp.	ss	65	6.15	0.58	1.08	0.17	57.45	57.45
BGT*	*—*	bg	55	0.26	0.05	0.56	0.10	13.75	71.20
Labridae	*Coris julis*	cj	65	0.19	0.06	0.49	0.08	10.63	81.83
Serranidae	*Serranus* spp.	se	75	0.18	0.04	0.04	0.02	4.01	85.84
Pomacentridae	*Chromis chromis*	ch	30	1.36	0.96	0.51	0.29	3.54	89.38
Sparidae	*Oblada melanura*	om	65	0.38	0.18	0.04	0.03	2.27	91.65
Sparidae	*Sarpa salpa*	sa	75	0.94	0.80	0.68	0.48	2.16	93.81
Mullidae	*Mullus* spp.	mu	90	0.02	0.01	0.08	0.03	2.14	95.95
Labridae	*Labrus* spp.	la	90	0.06	0.02	0.01	0.01	1.75	97.70
Sparidae	*Diplodus sargus*	ds	65	0.02	0.01	0.01	0.01	0.94	98.64
Sparidae	*Diplodus vulgaris*	dv	65	0.01	0.01	0.01	0.01	0.54	99.18
Labridae	*Thalassoma pavo*	tp	65	0.01	0.01	0.00	0.00	0.43	99.61
Scorpaenidae	*Scorpaena porcus*	po	65	0.01	0.01	0.00	0.00	0.26	99.87
Sphyraenidae	*Sphyraena* spp.	sy	100	0.01	0.01	0.00	0.00	0.13	100

**Figure 3:**
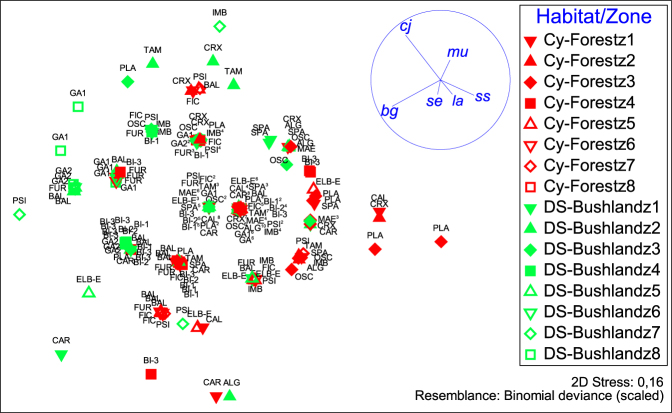
Non-metric MDS ordination plot of samples according to taxa abundances. For each sample of juvenile fish assemblage (i.e. each dot), levels of factors for the considered sample are displayed; Habitat: *Cystoseira* forest (Cy-Forest) in dark red *vs* Dictyotales-Sphacelariales bushland (DS-Bushland) in clear green; Zone: from z1 to z8, and Site. Site names of samples are given in upper case character on the chart next to the dot figuring the sample. When several samples for a same site and habitat coincide on the plot, number of samples is given in superscript Arabic number next to site name in order to make the chart clearer. Zones 1 to 4 belong to La Revellata locality (filled symbol) and zones 5 to 8 belong to Scandola locality (empty symbol) (see Fig. 1). Correlation vectors (Spearman) of juvenile abundance are showed for correlations >0.2, i.e. for *Symphodus* spp. (ss), *Coris julis* (cj), BGT (bg), *Serranus* spp. (se), *Mullus* spp. (mu), and *Labrus* spp. (la).

Definitions of y_0_ + y_+1_ size upper limits (mm, TL); “−“ = not defined in this study; Taxa abr. = taxa name abbreviations used in Fig. 3; Mean abundance (ind.m^−2^); Standard Error (se); Similarity analysis (SIMPER) of abundance data between groups of samples according to habitat DS-Bushland *vs C. balearica* forest (Average dissimilarity = 76,67%): individual (Contrib.) and cumulated (Cum.) contributions to group dissimilarity.

*BGT = Blenniidae-Gobiidae-Tripterygiidae.

### Study design and data analysis

A model was fitted to juvenile assemblage descriptors (i.e. multivariate density and univariate richness, total density and taxa-specific density) in order to test their responses to habitats, localities, zones and sites: factor habitat has two levels (*Cystoseira* forest and DS bushland) and is fixed; factor locality (lo) has two levels (Revellata and Scandola) and is random; factor zone (zo) has 4 levels (4 zones at both localities, see Fig. 1), is random and nested in Factor locality; factor sites (si) has 3 levels per zone at La Revellata (12 sites in total: SPA, CAR, CAL, CRX, TAM, ALG, MAE, OSC, PLA, BI1, BI2, BI3) and 2 levels per zone at Scandola (8 sites in total: BAL, ELE, FUR, FIC, PSI, IMB, GA1 and GA2), is random and nested in factor zone. In order to compare juvenile descriptors between levels of factors we performed multi- or uni-variate PERMutational ANalysis Of Variance (PERMANOVAs)^37^ on the model including terms and all interactions^38^. Terms were then pooled as suggested by Anderson *et al*.^39^. There was no specific hypothesis to test in relation with the putative effects of these random spatial factors (loc, zo and si). The stratified sampling design was used to increase the power of PERMANOVA analyses, by removing from the residual variance some portions of variances putatively explained by these random spatial factors, which are proxies for a large array of environmental variables (at present, impossible to disentangle) varying at various spatial scales in the study area. The factor ‘locality’ was intended to account for coarse scale environmental patterns, such as global current patterns that may affect larval supply (due to their pelagic dispersal stage) and possibly juvenile density as a consequence^6^. Within each locality, zones were intended to account for intermediate scale environmental variability, for instance the configuration of the stretch of coast and putative consequences for local ecosystem functioning (e.g. sheltered coast *versus* exposed one^3^). Sites within each zone were intended to account for fine scale environmental variability, for instance abiotic substrate composition and structure, which may directly and/or indirectly affect juvenile distribution patterns^27^. Finally, within each site, both forest and bushland habitats were randomly sampled in habitat patches so that replicates respective to each habitat were interspersed spatially and temporally. Resemblance measure matrixes were calculated from the initial data matrix containing for each sample the abundance of juveniles for each species. Analyses were based (for multivariate data) on the binomial deviance (scaled) dissimilarity measure or (for univariate data) on Euclidian distances^40^. P-values were obtained by 999 permutations of residuals under a reduced model. Monte Carlo P-values were considered when there were not enough possible permutations (<200). Effects on juvenile assemblage composition were illustrated through a multivariate exploratory approach using a non-metric multi-dimensional scaling (nMDS). nMDS represents samples as points in low-dimensional space in such a way that the relative distance apart of all points are in the same rank order as the relative dissimilarities of the samples^41^. For each taxa (specific abundances), correlations of taxa-specific density with the 2-D ordination plot of samples were plotted by displaying correlation vectors. Spearman correlation was used given its non-parametric properties. In addition, individual species’ contributions to the separation of sample groups were examined by SIMPER (Similarity percentage breakdown) routines^39,41^.

Among *Cystoseira* forests only, a second model was fitted to forest descriptors (depth, volume) in order to test their responses to localities, zones and sites (same levels and conditions as above). Forest volume and depth significantly differed among localities, zones and sites. Furthermore, forest depth and volume were not correlated (see results). Consequently, a third model was fitted to juvenile assemblage descriptors in order to test their responses to covariates depth and volume and to the factors locality, zone and site. We also analyzed taxa-specific density for the dominant benthic taxa of juveniles (see results): *Symphodus* spp. (i.e. *S. roissali*, *S. ocellatus*, and *S. tinca*), *Sarpa salpa*, Blenniidae-Gobiidae-Tripterygiidae (“BGT”), *Coris julis*, and *Serranus* spp. (i.e. *S. cabrilla* and *S. scriba*). *Chromis chromis* and *Oblada melanura* are less strictly associated with the benthic habitat^27^ and were not studied individually.

Due to the intrinsic variability of ecological data, tests were considered significant for p-values < 0.1. Data treatment and analysis were performed using the R 3.1.3 statistical software^42^ and PERMANOVA + add on package for PRIMER software^39,41^.

## Results

### Juvenile assemblages in forests *versus* bushland

Regarding juvenile total abundance, the interaction term between habitat and site (Supplementary Fig. S6) was significant (PERMANOVA, F = 2.33, p = 0.006). For 10 out of 20 sites, total abundance differed between habitats. In most cases, mean total density was higher in *Cystoseira* forest (mean ± s.e. = 9.59 ind.m^−2^ ± 1.39) than in DS bushland (3.51 ind.m^−2^ ± 0.59) (Supplementary Fig. S6a). Concerning juvenile richness, both interaction terms between habitat and zone, and between habitat and site were significant (PERMANOVA, respectively F = 6.78, p = 0.003 and F = 1.64, p = 0.088). For 4 out of 8 zones, and for 11 out of 20 sites, juvenile richness differed between habitats. In most of the cases it was significantly higher in *Cystoseira* forest than in DS bushland (pairwise tests, Supplementary Fig. S6b and S6c).

In both habitats, the most abundant juveniles observed belonged to the taxa *Symphodus* spp. (Table 1; Fig. 2 and Supplementary Fig. S4). To a lesser extent, the other abundant taxa were *Chromis chromis*, *Sarpa salpa*, BGT, *Coris julis*, *Oblada melanura*, and *Serranus* spp. (Fig. 2). Juvenile assemblage mean composition differed according to habitat (Fig. 3; Table 2; PERMANOVA, F = 7.93, p = 0.059), as well as to the random factors locality, zone and site (PERMANOVA, all p < 0.05). Interaction terms between habitat and locality and between habitat and site were not significant (Table 2; PERMANOVA, respectively F = 1.49, p = 0.308 and F = 1.29, p = 0.16). This means that juvenile composition differences between forest and bushland are consistent whatever the locality or site. Four taxa explained 86% of the average dissimilarity (76.67%) between sample groups *C. balearica* forest *versus* DS bushland (Table 1): *Symphodus* spp., BGT, *C. julis* and *Serranus* spp. Juvenile assemblages in *Cystoseira* forests were mainly characterized by high densities of *Symphodus* spp. and (relatively to bushland) higher densities of *Serranus* spp. On the other hand, they were characterized by relatively lower densities of *Coris julis* and BGT (crypto-benthic taxa). Inversely, DS bushland juvenile assemblages, compared to forest, were characterized by higher densities of *C. julis* and BGT and relatively low densities of *Symphodus* spp. (Figs 2 and 3, Table 1).

**Table 2:** PERMANOVA table of results: comparison of juvenile assemblage between habitats, localities, zones and sites.

Source of variation	df	MS	Pseudo-F	P(perm/MC)
Habitat ha	1	38.074	7.9322	0.056
Locality lo	1	18.899	5.011	0.024
Zone zo(lo)	6	3.8647	2.514	0.026
Site si(zo(lo))	12	1.5373	2.5641	0.001
haxlo	1	4.8	1.4948	0.308
haxzo(lo)	6	3.3126	4.2694	0.002
haxsi(zo(lo))	12	0.7759	1.2941	0.16
Residuals	240	0.59955		
Total	279			

Table gives degrees of freedom (df), Mean Squares (MS), calculated pseudo-F, and P-values (P). P-values were obtained by 999 permutations of residuals under a reduced model (perm) or through Monte Carlo test (MC, see methods).

When looking at taxa-specific density for *Symphodus* spp. (the most abundant taxa in the studied habitats), the interaction term between habitat and locality was significant (PERMANOVA, F = 12.03, p = 0.008) and therefore we performed new analyses separately for each locality. Both at La Revellata and Scandola, the habitat term was significant (PERMANOVAs, respectively F = 128.58, p = 0.028 and F = 25.64, p = 0.030). *Symphodus* spp. densities were higher in *C. balearica* forest than in DS bushland (Supplementary Figure S7). Throughout all of the La Revellata sites, this pattern was consistent: interaction term between habitat and site was not significant (PERMANOVA, F = 1.42, p = 0.199) and although densities varied within a given habitat the abundance pattern across habitats was similar. At the Scandola locality the habitat and site interaction term was significant (F = 5.78, p < 0.001) but there again *Symphodus* spp. densities were systematically higher in *Cystoseira* forest than in DS bushland. This significant interaction was probably due to one site (GA2) were densities of *Symphodus* in the forest were about one order of magnitude higher than at other sites (Supplementary Figure S7).

### Variability of juvenile fish assemblage among *Cystoseira* forests

Among *C. balearica* forests, the depth of replicates significantly differed between localities, zones and sites (Supplementary Figure S5; PERMANOVA, all p < 0.06) and ranged from 1.0 to 8.6 meters. *Cystoseira* forest height ranged from 7.0 to 20.0 cm (mean ± se = 13.1 cm ± 0.2) and forest cover ranged from 60% to 100% (mean ± se = 93.9% ± 0.8). The synthetic descriptor’forest volume’ ranged from 0.06 to 0.20 cm^3^. It significantly differed among zones and sites (PERMANOVAs, respectively F = 6.97, p = 0.004 and F = 4.00, p < 0.001) but not between localities (Supplementary Figure S5). Forest depth and volume were not correlated (Spearman rank correlation, |rho| < 0.2, p > 0.1). Accordingly, forest volume and depth were included as covariates in our models (see M&M and below).

Among *Cystoseira* forest replicates, overall juvenile assemblage composition did not differ significantly according to covariates depth or volume. A significant effect came from the random factors zone and locality (explaining respectively 9.2% and 8.8% of assemblage variability according to the PERMANOVA estimates of components of variations) (Table 3). When considering juvenile richness among the forest, depth effect was not significant and neither was the effect of locality, zone or site. The effect of forest volume on richness was close to the significance level (PERMANOVA; Supplementary Table S1). For total juvenile abundance, only the factor site had a significant effect (PERMANOVA; Supplementary Table S2) and explained 11.6% of its variability.

**Table 3:** PERMANOVA table of results: juvenile assemblage variability among *Cystoseira* forests according to covariates Depth, Volume and factors Locality, Zone and Site.

Source of variation	df	MS	Pseudo-F	P(perm)
Depth de	1	5.6188	1.7062	0.258
Volume vo	1	3.4112	2.3312	0.097
Locality lo	1	4.4984	2.6026	0.082
Zone zo(lo)	6	1.7872	2.3482	0.009
Site si(zo(lo))	12	0.76818	1.3553	0.108
Residuals	118	0.5668		
Total	139			

Table gives degrees of freedom (df), Mean Squares (MS), calculated pseudo-F, and P-values (P). P-values were obtained by 999 permutations of residuals under a reduced model.

**Figure 4:**
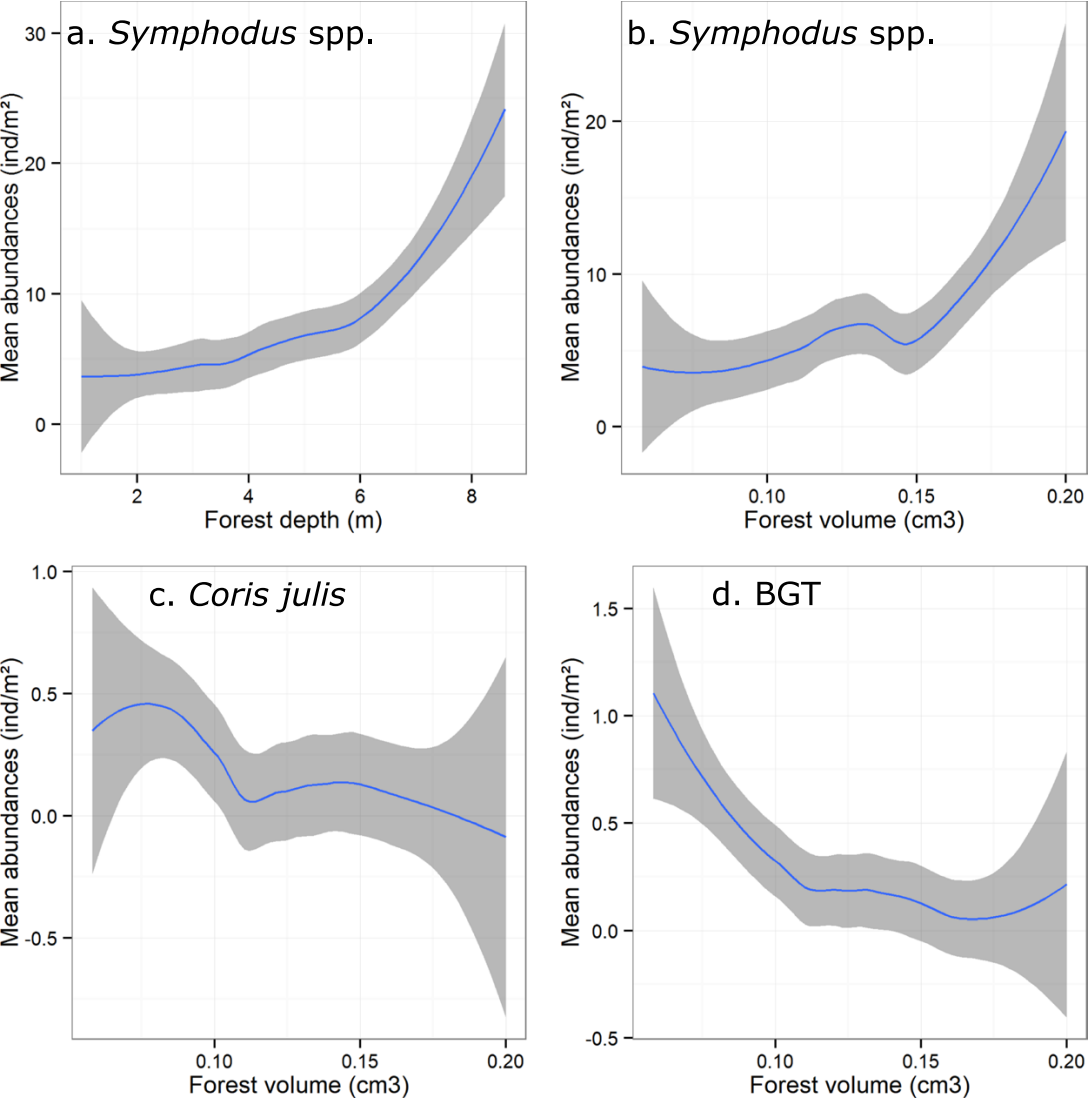
Smoothed curves of juvenile densities according to *Cystoseira* forest descriptors. For *Symphodus* spp. according to (**a**) depth (meters) and (**b**) volume (cm^3^). Same representation for (**c**) *Coris julis* and (**d**) BGT juvenile densities according to *Cystoseira* forest volume (cm^3^). Curves represent the mean and shadow areas of curves represent standard error (SE).

Significant patterns appeared when looking at the taxa-specific densities for the dominant taxa. For *Symphodus* spp. densities, both covariates depth and forest volume had a significant (and independent) effect (PERMANOVA, respectively F = 13.2, p = 0.017 and F = 18.9, p < 0.001; Supplementary Table S3; Fig. 4a,b). On one hand, forest volume explained 11.5% of *Symphodus* juvenile density variability, which were up to five-fold more abundant in thicker forests (Fig. 4b). On the other hand, depth explained 16.3% of this variability and juveniles were more abundant in deeper forests. Finally, the factor site explained 15.3% of *Symphodus* juvenile density variability (PERMANOVA, F = 3.50 and p < 0.001; Supplementary Table S3). Forest volume (but not depth) also significantly explained part of *Coris julis* and BGT juvenile density variations (respectively 2.9 and 8.8%). In contrast to *Symphodus* spp., *Coris julis* and BGT tended to be more abundant in sparser forests (PERMANOVAs; Supplementary Table S3; Fig. 4c,d). For these taxa another part of the density variability was significantly explained by locality (for *C. julis*) and zone (for BGT) (respectively 8.5% and 8.2%) (PERMANOVAs; Supplementary Table S3). For *Sarpa salpa* and *Serranus* spp. juvenile densities, none of the factors had a significant effect (PERMANOVAs, all p > 0.1).

## Discussion

In temperate marine environments, previous studies on juvenile fish assemblages have mainly focused on shallow rocky bottoms, estuaries or seagrass meadows^31,43–45^. Rocky reefs with marine algae forests have also been studied with regard to adult and juvenile fishes, particularly in the Mediterranean^27,46,47^. However, few studies have focused on *Cystoseira* (Fucales) forests^21,48^. To date, the spatial variability of juvenile assemblages among macrophytes habitats and its link with habitat descriptors has been investigated for seagrass meadows^45,49,50^, but rarely for Fucales macro-algae forests^16^ and even less so in the Mediterranean. The present study, through a large spatial scale sampling effort, (1) has demonstrated that fish juvenile assemblage differed between arborescent *C. balearica* forests and Dictyotales – Sphacelariales bushland, and that this pattern was consistent through space. (2) Secondly, among *Cystoseira* forests, juvenile assemblage variability was explained by forest descriptors (notably variations in canopy structure). (3) Thirdly, some residual spatial variability of juvenile assemblages may remain due to other un-controlled drivers.

Forested *versus* non-forested habitats differed in terms of juvenile assemblage descriptors. *C. balearica* forests hosted more abundant and richer juvenile assemblages than DS bushlands and they differed in terms of taxa composition. These differences were consistent through space at scales of 1, 10 and 40 km (between sites, zones or localities). For *Symphodus* spp. juveniles, abundance patterns we observed here (i.e. [*C. balearica*] > [DS bushland]) confirmed a previous study carried out at a single site (ELE) in 2009^21^. In contrast to the *Symphodus* spp. results, *C. julis* juveniles were more associated with Dictyotales-Sphacelariales bushland or among *Cystoseira* forests displaying sparser structure of the canopy, i.e. with some cover of Dictyotales-Sphacelariales. Thus, presence or absence of the erect macrophyte canopy (here *Cystoseira*) had a dramatic influence on juvenile fishes. Previous studies highlighted the higher density of some juvenile taxa among the canopy forming *Posidonia oceanica* meadows (*Symphodus ocellatus*, *Symphodus mediterraneus*, *Serranus cabrilla*, *Diplodus annularis*, *Spondyliosoma cantharus* and *Sarpa salpa*) particularly in comparison with sandy bottoms or rocky DS bushland^46,51^. Other authors suggested that differences in fish species richness and abundance are primarily related to habitat structure, e.g. the three-dimensional structure of the canopy forming *Posidonia* meadows (e.g. heterogeneity)^50^.

The three-dimensional structure of the habitat had a significant influence in our data. Because of our large spatial scale sampling effort, we were able to demonstrate that part of the spatial variability of juvenile densities in a given habitat (i.e. among *Cystoseira* forests) was correlated (positively or negatively, according to taxa) with habitat abiotic and biotic descriptors (notably small-variations in canopy volume). Other works in temperate seas have also reported on the influence of habitats formed by macrophytes and their three-dimensional characteristics. In northeast New Zealand, within the habitat composed by the canopy forming Phaeophyceae *Ecklonia* and *Carpophyllum*, Jones^52^ showed that mean juvenile densities (*Pseudolabrus celidotus*, Labridae) increased exponentially with the mean weight of algae per square meter. This significant effect was even stronger in shallow (<8 m) than in deeper (>8 m) areas. In addition, in the same study, a macro-algal removal experiment resulted in significantly lower juvenile fish settlement (expressed as juvenile abundance per surface unit). This was confirmed by the observation of increased settlement after the recovery of an algal forest over a previously barren rocky reef.

What are the underlying processes? It is important to quantify the processes driving the variability in juvenile densities within a given juvenile habitat (here among *Cystoseira* forests) or density patterns across habitats (here in forest *versus* bushland). It may be explained by the different levels of three-dimensional structure in different habitats (or degraded facies of a given habitat). This determines their quality for juveniles, sensu Hindell *et al*.^53^, i.e. by affecting the ratio of food availability to the predation rate resulting in the active choice and/or differential mortality of juveniles between habitats^4,9^. During *ex-situ* experiments, Thiriet^54^ observed active micro-habitat choices for juveniles of *Symphodus* spp. which were modulated by the type of predator present and the differential predation success of *Serranus* spp. on these juveniles according to the habitat complexity (arborescent forest *vs* bushland). In that case, more complex habitat, which offered more refuge, lowered predation success. Nevertheless, caution should be shown in drawing such conclusions since greater structural complexity of macrophytes among *in-situ* natural habitats does not always inhibit predation, but it may change predator behavior or the types of predators present^55^. Consequently, a full understanding of these underlying processes still requires further study.

Accordingly, the transformation of juvenile habitat (e.g. reduction of *Cystoseira* canopy density or patch-size) may strongly affect the recruitment of several species of littoral fishes. In order to further test this hypothesis, artificial *Cystoseira* thalli were used as an accurate method to experimentally manipulate *in-situ* habitats^21^. Critical threshold levels of forest depletion for fishes^45^ should be further investigated. Nevertheless, here we observed that different species had contrasting responses to forest structure descriptors (e.g. *C. julis versus Symphodus* spp.). At the scale of the seascape (i.e. the mosaic of various habitats), the complementarity of various coexisting juvenile habitat configurations (e.g. levels of density) or types (e.g. forest *vs* bushland) (i.e. a heterogeneous seascape mosaic) may satisfy a greater number of species than a single homogenous habitat covering the equivalent area. This is predictable according to the concept of spatial partitioning of juveniles of various species (or of various size classes) within various habitats as described for Sparidae in previous studies^3,31^. In our study, *Serranus* spp. juvenile densities were higher in the forest but their spatial variability could not be explained by forest descriptors. Previous work has suggested that they may be associated with ecotones (i.e. borders between habitat types)^54^ and accordingly to the’edge effect’. This’edge effect’ may result from the complementarity of resources specific to each habitat. At ecotones, individuals may alternatively use the optimum habitat corresponding to the resource needed (e.g. food or shelter)^9,55^. In contrast, seascape homogenization (i.e. dominance of a single habitat) will probably be detrimental to fish recruitment as observed, for example, in *Caulerpa taxifolia* meadows^36,56^.

Which other factors may explain the residual spatial variability of juveniles? In the present study, after incorporating the variability of juvenile assemblage descriptors due to forest descriptors, some spatial variability of assemblages remained between sites, zones and/or localities. Therefore, although the three-dimensional characteristics of a macro-algae forest may explain part of juvenile density patterns, substantial spatial variability remains both at local (<1 km) and regional (40 km) scales. This is consistent with previous studies in other macrophytes-formed habitats or in other habitats. For example, for *Diplodus* spp. juveniles Vigliola *et al*.^34^ showed a strong inter-annual and spatial variability of these juvenile densities in nurseries at 20 sites dispersed along rocky shores in the northwestern Mediterranean basin. This residual spatial variability may be explained by other factors such as oceanographic currents^57^ or coast morphology^3^ that shape larval dispersal, the input of settlers in nurseries and consecutive juvenile density patterns in space. This residual variability should not be neglected since it has conservation implications^6^. Juvenile habitats must be protected not only in one place but rather in a network of spatially dispersed places in order to prevent local failure in settlement. Both the study and the management of fish essential habitats and assemblages must take into account a nested spatial scale of analysis and adopt a ’seascape approach’^58^.

In the present study, juvenile density was also partly related to abiotic factors such as small variations in depth. Milazzo *et al*.^59^ highlighted the contrasted vertical distribution of two wrasse species. Cuadros^25^ also found that labrid juvenile densities were correlated with depth. Spatial differences in juvenile densities or in juvenile mortality rates have previously been explained by adult conspecific and predator density spatial distributions^60^. Similarly, the depth distribution of juveniles, for a given habitat, may also be shaped by the spatial distribution of adult conspecifics and predator densities, which in turn are influenced in particular by protection levels^60^. Further studies should therefore analyze juvenile density patterns within *Cystoseira* forests according to both depth and protection levels (no-take area *versus* non-protected).

Furthermore, in the case of crypto-benthic taxa (Blenniidae, Gobiidae, Triperygiidae), our results suggest that their juveniles were more abundant in the DS-bushland or in the sparser forests *versus* denser ones. This is in contradiction with another recent study^48^ which found greater biomass of these taxa in the forested habitat. This is probably due to our sampling method (visual census) which may have underestimated crypto-benthic taxa abundances due to their camouflage and behavior. Thiriet *et al*.^48^ used a novel method based on Enclosed Anaesthetic Station Vacuuming which appears to be a promising complement to classical UVC. On the other hand, for most of the species presented here (i.e. necto-benthic taxa), our sampling method was well-suited to our aims since these species are sedentary when juvenile^27,28,35,61^, ruling out significant movements between adjacent sites. In previous studies, similar size (from 1 to 10 m²) and numbers of replicates (from 6 to 10 per site) were proven to provide accurate juvenile density data^17,21,24,25,48^. In our study, sampling units of such size (1 m²) were necessary and were chosen specifically because most of studied juveniles (sized about 10 mm TL at settlement) may be shy and their observation requires a vigilant observer focused on small surface areas^61^. Otherwise, densities would be underestimated. For the few mobile (transient) juvenile species (e.g. *Sphyraena* spp.), the time spent for each replicate (5 min) seemed sufficient to cope with the surface area of the sampling unit and to encompass movement of species. In this sense, the density measure obtained may be interpreted as a measure of a flux, rather than an absolute density. In any case, in the present study, this does not impair comparisons between habitats, such as those undertaken here. Nevertheless, as a basis for better understanding of the settlement and recruitment dynamic of taxa such as *Sphyraena* spp., specific studies remain scarce^62^ and further work is still required. As a conclusion, future studies using UVC for juveniles may make use of improvements to traditional underwater census techniques to better account for differences in behavior among and within species: Prato *et al*.^63^ suggested combining surveys with multiple sampling unit surface areas in order to allow for a more accurate fish assemblage assessment.

## Conclusion

In conclusion, juvenile fish differed between the arborescent, canopy-forming *Cystoseira* habitat *versus* the structurally more simple Dictyotales – Sphacelariales bushland. Forests hosted richer and three-fold more abundant juvenile assemblages. This was consistent through space at nested spatial scales from 1 to 40 km along NW Mediterranean subtidal rocky shores and confirmed preliminary results obtained during another study. Moreover, among *Cystoseira* forests, juvenile assemblage descriptors varied through space and this was partly explained by forest characteristics (notably the three-dimensional structure of the canopy). Further studies are needed to explain the other residual part of the spatial variability of juvenile assemblages since it may have conservation implications. Finally, we demonstrated that macro-algae forests are important juvenile habitats, although further studies are needed to describe the definitive contribution (i.e. the’nursery value’) of each habitat in the seascape to the final recruitment into the adult population^43^. For this purpose, additional work on juvenile survival, growth and movement towards adult habitats, in particular, is required.

## Electronic supplementary material

Supplementary information
