# Supplementary material for: Juvenile fish assemblages in temperate rocky reefs are shaped by the presence of macro-algae canopy and its three-dimensional structure
Source: Sci Rep. 2017 Nov 7;7:14638. doi: 10.1038/s41598-017-15291-y (PMC5676688; doi:10.1038/s41598-017-15291-y)
Supplement: Supplementary file 1 — Supplementary information [file 41598_2017_15291_MOESM1_ESM.pdf]

Title:
Juvenile fish assemblages in temperate rocky reefs are shaped by the presence of macro-algae canopy and its three-dimensional structure

Contributors:
Adrien Cheminée, Jérémy Pastor, Olivier Bianchimani, Pierre Thiriet, Enric Sala, Jean-Michel Cottalorda, Jean-Marie Dominici, Pierre Lejeune and Patrice Francour

Supplementary information file

Note : for each PERMANOVA, table of results gives degrees of freedom (df), Mean Squares (MS), calculated pseudo-F, and P-values (P). P-values were obtained by 999 permutations of residuals under a reduced model (perm) or through Monte Carlo test (MC, see methods).

Supplementary Table S1. PERMANOVA table of results: juvenile richness among *Cystoseira* forests

Source of variation	df	MS	Pseudo-F	P(perm)
Depth de	1	0,48676	0,16633	0,709
Volume vo	1	5,2055	3,4788	0,087
Locality lo	1	3,8197	2,0892	0,169
Zone zo(lo)	6	1,8869	2,2136	0,125
Site si(zo(lo))	12	0,86249	1,4921	0,119
Residuals	118	0,57805		
Total	139			

P-values were obtained by 999 permutations of residuals under a reduced model

Supplementary Table S2. PERMANOVA table of results: juvenile total abundance among *Cystoseira* forests

Source of variation	df	MS	Pseudo-F	P(perm)
Depth de	1	251,85	1,6055	0,268
Volume vo	1	771,56	2,4098	0,133
Locality lo	1	14,52	4,04E-02	0,845
Zone zo(lo)	6	359,68	0,81394	0,578
Site si(zo(lo))	12	449	1,8162	0,044
Residuals	118	247,22		
Total	139			

P-values were obtained by 999 permutations of residuals under a reduced model

Supplementary Table S3. PERMANOVA tables of results for taxa-specific juveniles abundance among *Cystoseira* forests

a. *Symphodus* spp. juvenile abundance among *Cystoseira* forests

Source of variation	df	MS	Pseudo-F	P(perm)
Depth de	1	1191.5	13.194	0.017
Volume vo	1	828.85	18.886	0.001
Locality lo	1	119.81	2.9295	0.125
Zone zo(lo)	6	37.725	0.4261	0.876
Site si(zo(lo))	12	90.816	3.4957	0.001
Residuals	118	25.979		
Total	139			

b. *Coris julis* juveniles abundance among *Cystoseira* forests

Source of variation	df	MS	Pseudo-F	P(perm)
Depth de	1	0.21433	9.40E-02	0.767
Volume vo	1	2.408	4.5152	0.044
Locality lo	1	3.5597	10.474	0.018
Zone zo(lo)	6	0.32149	0.51952	0.768
Site si(zo(lo))	12	0.62669	1.5555	0.096
Residuals	118	0.40288		
Total	139			

c. Blenniidae-Gobiidae-Tripterigiidae juveniles abundance among *C.* forests

Source of variation	df	MS	Pseudo-F	P(perm)
Depth de	1	2.0976	5.1751	0.075
Volume vo	1	4.8674	8.8188	0.004
Locality lo	1	0.25092	0.33872	0.566
Zone zo(lo)	6	0.77441	3.6673	0.031
Site si(zo(lo))	12	0.20824	0.71471	0.755
Residuals	118	0.29137		
Total	139			

P-values were obtained by 999 permutations of residuals under a reduced model

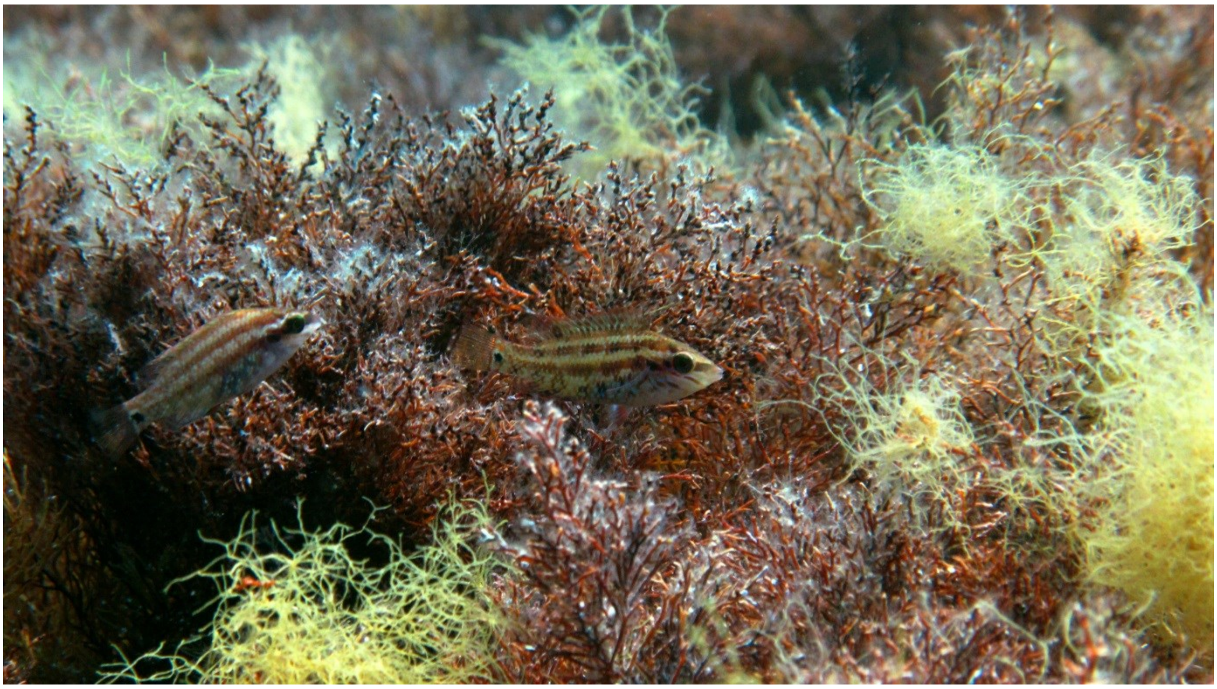

Supplementary Figure S4. *Symphodus roissali* juveniles (about 45 mm TL) among a *Cystoseira brachycarpa* var. *balearica* forest (3m depth, La Revellata Bay, Corsica, July 16th 2010). Picture by Adrien Cheminée.

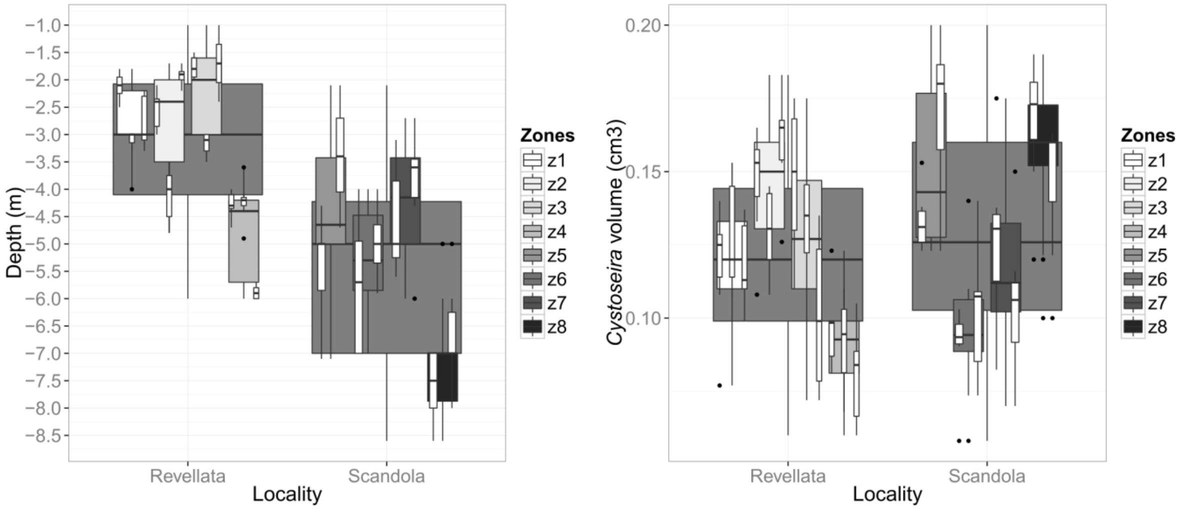

Supplementary Figure S5. Tuckey boxplots of *Cystoseira* forest descriptors: Depth (meters) and volume (cm^3) – boxes of different shapes display levels of factors locality (Revellata *vs* Scandola), zones (z1 to z8, in scale of greys) and sites (white filled boxes). Box plots indicate the median (bold line near the center), the first and third quartile (the box), the extreme values where distance from the box is at most 1.5 times the inter quartile range (whiskers), and remaining outliers (dark circles).

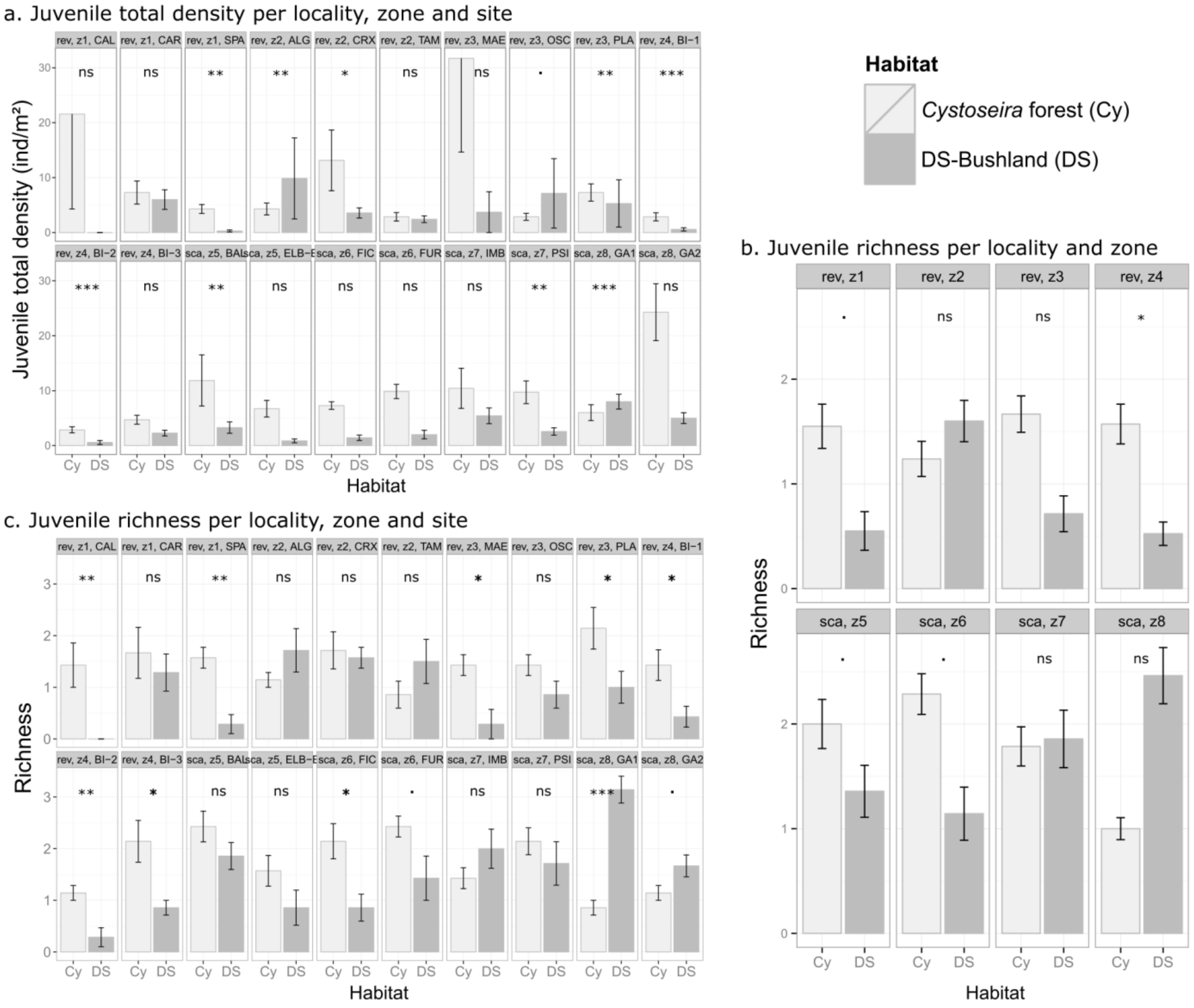

Supplementary Figure S6. Barplots of juvenile total density and richness within both studied habitats: *C. balearica* forests (Cy) and Dictyotales-Sphacelariales (DS) bushland. Mean and standard error (error bars) are given. (a) Habitat term for total density in each of the 20 sites among our study zones (z1 to z8) from both localities (Revellata "rev" and Scandola "sca"). (b) & (c) Habitat term for richness in each zone and site (see Fig. 1). Pairwise test results are given ("ns": not significant; "**.**": P < 0.1; "*": P < 0.05; "**": P < 0.01; "***": P < 0.001.). P-values were obtained by 999 permutations of residuals under a reduced model.

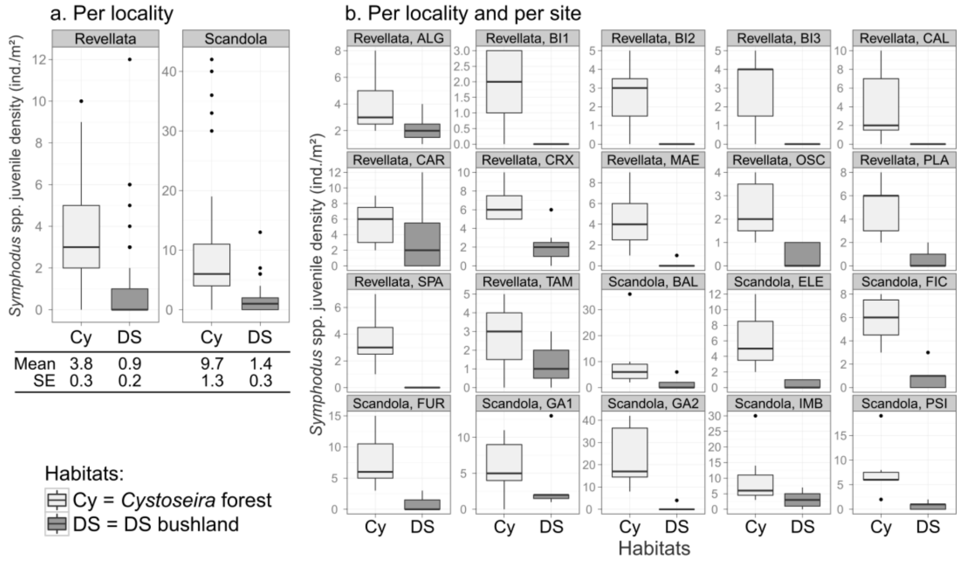

Supplementary Figure S7. Tuckey boxplots of *Symphodus* spp. densities within both studied habitats. (a) Habitat term for both localities (see PERMANOVAs results in the text), means and standard errors (SE) are given. (b) Habitat term for each site per locality (see sites localization on Fig. 1). Density differences between habitat levels were systematically significant (all pair-wise tests: $P<0.05$). Box plots indicate the median (bold line near the center), the first and third quartile (the box), the extreme values where distance from the box is at most 1.5 times the inter quartile range (whiskers) and remaining outliers (dark circles).
